# The Rise of Health Economics: Transforming the Landscape of Economic Research

**DOI:** 10.1002/hec.70044

**Published:** 2025-09-24

**Authors:** Lorenz Gschwent, Björn Hammarfelt, Martin Karlsson, Mathias Kifmann

**Affiliations:** ^1^ University of Duisburg‐Essen Duisburg Germany; ^2^ Swedish School of Library and Information Science (SSLIS) University of Borås Borås Sweden; ^3^ CINCH University of Duisburg‐Essen Essen Germany; ^4^ Hamburg Center for Health Economics University of Hamburg Hamburg Germany

## Abstract

This paper explores the evolving role of health economics within economic research and publishing over the past 30 years. Historically, largely a niche field, health economics has become increasingly prominent, with the share of health economics papers in top journals growing significantly. We aim to identify the factors behind this rise. Using a combination of bibliometric methods and natural language processing (NLP), we classify abstracts to define health economics. Adapting NLP methods to evaluate the novelty, impact, and quality of academic papers, we demonstrate that the mainstreaming of health economics is driven by innovative, high‐quality research, with two notable waves in quality ratings that highlight the emergence and impact of distinct subfields within the discipline. We find a strong positive correlation between citations and quality ratings, with health economics papers receiving fewer citations for their quality compared to other economics fields. Pandemic‐related research received a high number of citations during 2020 and 2021; however, our findings indicate that this work was not systematically more novel or impactful than prior studies within the same subfield.

## 1 Introduction

This article explores the evolving role of health economics within general economic research and publishing over the past 30 years. Historically, despite seminal contributions from scholars like Arrow (1963) on medical care economics, Newhouse (1970) on hospitals, Grossman (1972) on health production and the demand for health, and advances in econometric techniques (Newhouse 1987), health economics remained largely a niche field. It rarely featured in the most prestigious general‐interest economics journals.

In recent decades, the situation has changed dramatically. Health economists have increasingly entered the mainstream, and leading economists have increasingly turned their attention to health‐related topics. Health economics is one of the fastest‐growing fields within economics (Bornmann and Wohlrabe 2024). According to our analysis, the share of health economics papers in “top‐5” journals grew from 2% to 6%, while their presence in other general interest journals doubled from 7% to 14% between the mid‐1990s and 2020. Even journals focused on distinct though related fields (labor, development, public economics) saw a quadrupling of health economics papers during this period (see also Mitra et al. 2020).

In this paper, we seek to understand whether the rise of health economics research is attributable mainly to disruptive, innovative research that represents a break from the past and leaves a lasting impact—or rather to conventional research which excels in the current state of the art. We build on a method proposed by Kelly et al. (2021) to measure the *novelty*, *impact*, and *quality* of patents, based on their similarity with earlier and later patents. “Novelty” captures low backward similarity, “impact” captures high forward similarity, and “quality” is a combination of novelty and impact. We adjust this method to make it suitable for research articles.

A key contribution of our analysis is a new approach to identifying what constitutes a health economics paper. Previous studies by Rubin and Chang (2003) and Wagstaff and Culyer (2012) used JEL codes. However, this approach faces a number of limitations, for example, a paper can be assigned multiple JEL codes across different fields, or codes may be added to a field despite having little relevant content. The methodological contribution of our paper is to develop a novel approach harnessing advances in natural language processing (NLP). Using RoBERTa (Liu et al. 2019), a transformer‐based large language model, we classify abstracts to determine if they belong to health economics. Our final classification combines two independent approaches, one representing the idea that health economics is what the dedicated field journals publish (as opposed to journals from other fields), and one representing the idea that health economics is research conducted by health economists. We show that this combined approach outperforms either method alone.

Our main finding is that, on average, health economic papers rate consistently higher on “quality” than papers from other fields, suggesting that their rise is driven by innovation rather than conformity. Additionally, we identify two significant waves in the “quality” ratings of health economics research—first between 2006 and 2009, and again from 2014 to 2016—which are distinct from other economic fields. We are able to show that distinct subfields contribute to these “quality” booms. We further explore the relationship of our “quality” ratings to citations. We find significant positive correlations, which are considerably higher if we condition on the journal and the year in which the papers were published. Our analysis also shows that health economics papers are systematically less rewarded by citations for “novelty”, “impact”, and “quality” compared to papers from other fields of economics. Finally, we examine to what extent COVID‐related research contributed to the increasing importance of health economics within the economics literature. We find a spike in citations of this research, but not in our “quality” ratings.

The paper is organized as follows. The next section presents the methods we use for paper classification. Section 3 defines our innovation measures and presents how these evolve over time. We analyze the relationship of our indicators to citations, compare the performance of health economics and other economics papers, and explore the impact of COVID‐related research. Section 4 concludes.

## 2 Paper Classification

### 2.1 Analysis Sample

The analysis sample and the data sample that provide the training data for classifications consist of the universe of articles (excluding non‐research contributions) published in 25 prominent economics journals over the 1994–2023 period. The sample of journals was selected according to the following principles:

1. Five journals identified as the most prominent field journals of health economics according to Hammarfelt and Karlsson (2023): Health Economics (HE), Journal of Health Economics (JHE), American Journal of Health Economics (AJHE), European Journal of Health Economics (EJHE), International Journal of Health Economics and Management (IJHEM).
2. Prestigious journals with a remit that includes health: American Economic Review (AER), Journal of Political Economy (JPE), Quarterly Journal of Economics (QJE), Econometrica (ECMA), Review of Economic Studies (ReStud), American Economic Journal: Applied Economics (AEJ: Applied), American Economic Journal: Economic Policy (AEJ: Ec Policy), Review of Economics and Statistics (RESTAT), Journal of the European Economic Association (JEEA), Economic Journal (EJ), Journal of Human Resources (JHR), Journal of Public Economics (JPubE), RAND Journal of Economics (RAND), Journal of Development Economics (J Dev Econ), Journal of Labor Economics (JoLE).
3. Field journals for non‐health fields: American Economic Journal: Macroeconomics (AEJ: Macro), JoLE, JPubE, Journal of Economic Growth (J Econ Growth), J Dev Econ, Theoretical Economics, Journal of International Economics, Journal of Econometrics.

The idea underlying this selection is that the analysis sample should include all potential outlets for health economic research that are at least as prestigious within the profession as the top field journals HE and JHE. This is the motivation for including category (2). In addition, a journal‐based classification of abstracts requires including journals from other fields, in particular fields that are related to health economics (so as to avoid false positives). This is the motivation behind category (3).

The sample, encompassing all 38,116 research papers in these 25 journals during the 1994–2023 period, was accessed from *Web of Science* on March 25, 2024 (Clarivate 2024).

### 2.2 Method

Operationalizing fields and specialties in bibliometrics is a challenging task. The most common approach is to base it on specific journals due to its straightforwardness (cf. Mitra et al. 2020; Hammarfelt and Karlsson 2023). However, this approach is not viable in our case since we aim to study the impact of health economics on general academic publishing in economics. Instead, bibliometric studies of economics often rely on the JEL classification of papers. One challenge is that the JEL classification aims to categorize both economists and their output for a wide range of stakeholders, including researchers, publishers, recruiters, and various external entities. The codes have resulted from numerous influences, external demands, and differing visions of the discipline (Cherrier 2017). Therefore, the classification may deviate from what is optimal from a bibliometric point of view. For example, the JEL codes for a single paper may span multiple fields. Besides, the ordering of JEL codes is inconsistent over time (cf. Angrist et al. 2020), and JEL codes for a certain field may be added despite having negligible content related to that field (Wagstaff and Culyer 2012). Kosnik (2018) reports that even though the various JEL codes assigned to papers broadly reflect their contents, there is a striking disagreement between editors and authors regarding the appropriate JEL classification of papers.

Despite the known issues with JEL codes, researchers rarely explore alternative approaches. Some alternatives include using keywords to define a field (Geiger 2017) or identifying the corpus of significant papers based on review articles (Braesemann 2019). A recent study in economic history incorporated information from abstracts and main texts in addition to JEL codes, highlighting the limited reliability of the JEL classification as a key motivation (Cioni et al. 2023).

In this paper, we utilize recent advances in natural language processing (NLP) to classify economics publications into health economics or other sub‐fields based on their titles and abstracts. Specifically, we employ RoBERTa, a large language model (LLM) based on the transformer architecture, known for its high performance in text classification tasks (Liu et al. 2019; Vaswani et al. 2023). While the application of LLMs in economics has been limited due to concerns about performance on longer texts and interpretability (Ash and Hansen 2023), recent studies have successfully used LLMs for classification tasks in economics, such as job postings (Hansen et al. 2023) and social media posts (Gehring and Grigoletto 2023).

To classify health economics papers, we created two labeled datasets. Each dataset allows us to approach the classification of health economics publications as a binary classification problem with the label “1” representing health economics and the label “0” representing other economics publications. The first dataset, denoted *journal‐based classification*, labels papers published in health economics journals as health economics and those in other fields (category 3 above) as non‐health economics. This dataset includes 6339 health economics papers out of a total of 19,434 papers.

Defining a field based on field journals is common in bibliometric research (cf. Hammarfelt and Karlsson 2023). However, one potential concern is that titles and abstracts in general‐interest journals may be different in style from titles and abstracts in field journals, leading to poor external validity. To address this, we consider a second approach, termed *author‐based classification*. This approach entails two steps: first, we use the field journals (categories 1 and 3 above) to classify authors as either health economists or non‐health economists, depending on whether more than 50% of their publications are in health economics journals. We then turn to the general interest journals (category 2, excluding the field journals listed in category 3) and label papers authored by health economists as health economics. This dataset comprises 498 health economics papers out of a total of 12,464 papers.

On both datasets, we trained a classifier based on RoBERTa‐large (see Appendix A for details). The combined classifier was then used to predict health economics papers in a total sample of 38,116 papers.

### 2.3 Results

Table 1 presents the statistics of how the classifiers performed in different training data samples, including journal‐based and author‐based samples, as well as in the combined dataset.

**TABLE 1:** Combining statistics—performance.

	Journal sample (N=20,440)			Author sample (N=7,053)			Combined (N=27,493)		
Criterion	Sensitivity	Specificity	F1	Sensitivity	Specificity	F1	Sensitivity	Specificity	F1
$p_{J}>0.500$	0.960	0.969	0.956	0.481	0.956	0.441	0.932	0.964	0.923
$p_{J}>0.327$	0.961	0.969	0.956	0.483	0.956	0.442	0.933	0.964	0.923
$p_{A}>0.500$	0.960	0.953	0.943	0.708	0.934	0.513	0.945	0.946	0.909
$p_{A}>0.828$	0.943	0.963	0.941	0.672	0.948	0.537	0.926	0.957	0.912
$p_{C}>0.498$	0.972	0.967	0.960	0.581	0.954	0.501	0.949	0.962	0.929

*Note:* The table reports the classification performance of the two RoBERTa classifiers. $p_{J}$ denotes the prediction probability of the *journal‐based* classifier, $p_{A}$ is the probability of the *author‐based* classifier, and $p_{C}$ represents the *average* of the two. We report the standard test properties, sensitivity and specificity, along with the F1 score $\left(F1=\frac{2TP}{2TP+FP+FN}\right)$, where TP represents the true positive rate, FP the false positive rate, and FN the false negative rate (Van Rijsbergen 1979).

The classifiers demonstrated excellent performance in the journal‐based training data sample (left panel), with both sensitivity and specificity exceeding 0.95. However, sensitivity in the author‐based sample (middle panel) was notably lower, reflecting the noisier nature of an author's identity as a signal for classification. Nevertheless, when the two samples are combined (right panel), the overall performance is very high in general. To ensure robust classification across all sub‐samples, we use the global F1 score (rightmost column) as our main criterion. We find that averaging the test statistics and applying a cutoff of 0.498 yields the highest global F1 score. Thus, we implement this combined classifier in subsequent analyses, which results in 8686 health economics papers out of 38,116 total papers in our sample.

**FIGURE 1:**
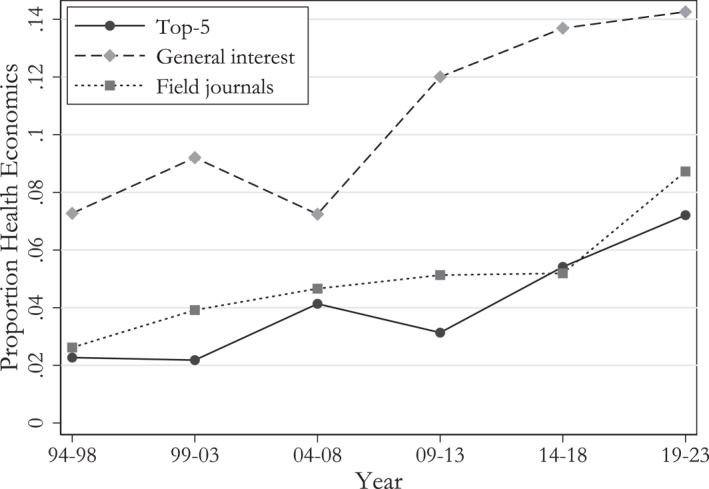
Proportion of health economics papers by type of outlet. “Top‐5” includes AER, JPE, QJE, REStud, and Econometrica; “general interest” includes REStat, AEJ: Applied, AEJ: Economic Policy, EJ, JEEA, RAND Journal, JHR; “Field journal” includes J Dev Econ, J Econ Growth, J Econometrics, J Int Econ, JOLE, JPubE. Papers are labeled as “health economics” based on the classification algorithm presented in Section 2.2.

Figure 1 shows our results by type of outlet. We observe a rising proportion of health economics papers in various types of journals. Between the mid‐1990s and 2020, the share of health economics papers in “top‐5” journals grew from 2% to 6%, while their presence in other general interest journals doubled from 7% to 14%. Even journals focused on distinct though related fields (labor, development, public economics) saw a quadrupling of health economics papers during this period.

In order to give a flavor of how the classification works, Table 2 provides examples of papers from six different groups, defined by how confidently they are classified as either health or non‐health papers. In this case, confidence is operationalized as the distance of the prediction probability from the cut‐off.

**TABLE 2:** Examples of classifications by degree of confidence.

Reference	Journal	Title	Score
I. Health economics, high confidence			
Manning and Marquis (1996)	JHE	Health insurance: The tradeoff between risk pooling and moral hazard	0.999
Decker (2005)	JHR	Medicare and the health of women with breast cancer	0.999
Coile et al. (2014)	AEJ: EP	Recessions, older workers, and longevity: How long are recessions good for your health?	0.999
II. Health economics, medium confidence			
Baines and Whynes (1996)	HE	Selection bias in GP fundholding	0.998
Rellstab et al. (2020)	JHE	The kids are all right—labor market effects of unexpected parental hospitalizations in The Netherlands	0.998
Sabariego et al. (2011)	EJHE	Cost‐effectiveness of cognitive‐behavioral group therapy for dysfunctional fear of progression in cancer patients	0.998
III. Health economics, low confidence			
Reisinger et al. (2019)	JHE	Parallel imports, price controls, and innovation	0.529
Chari et al. (2017)	JDevEc	The causal effect of maternal age at marriage on child wellbeing: Evidence from India	0.525
Gundersen and Kreider (2008)	JHR	Food stamps and food insecurity: What can be learned in the presence of nonclassical measurement error?	0.519
IV. Not health economics, low confidence			
Persico and Venator (2021)	JHR	The effects of local industrial pollution on students and schools	0.167
Boomhower (2019)	AER	Drilling like there's no tomorrow: Bankruptcy, insurance, and environmental risk	0.152
Knittel (2004)	RESTAT	Regulatory restructuring and incumbent price dynamics: The case of US local telephone markets	0.140
V. Not health economics, medium confidence			
Gollin et al. (2016)	J econ growth	Urbanization with and without industrialization	0.002
Aradillas‐Lopez and Rosen (2022)	J econometrics	Inference in ordered response games with complete information	0.002
Mintz and Smart (2004)	JPubE	Income shifting, investment, and tax competition: Theory and evidence from provincial taxation in Canada	0.002
VI. Not health economics, high confidence			
Alvarez‐Cuadrado et al. (2004)	J econ growth	Habit formation, catching up with the joneses, and economic growth	0.001
Sampson (2016)	QJE	Dynamic selection: An idea flows theory of entry, trade, and growth	0.001
Krusell et al. (2010)	ECMA	Temptation and taxation	0.001

*Note:* “High confidence” corresponds to percentile 100 of confidence within each classification, “Medium confidence” corresponds to percentile 51 and “Low confidence” corresponds to percentile 5.

The results reported in Table 1 suggest that our classification has excellent internal validity. We also consider the agreement between our classification and other attempts at classifying economics papers. In this part, we draw upon the work of Angrist et al. (2020), who classified economics papers published in the 1970–2015 period into different fields based on their JEL codes. There are 23,906 papers in both datasets, of which 2112 are classified as health economics according to our approach.

**FIGURE 2:**
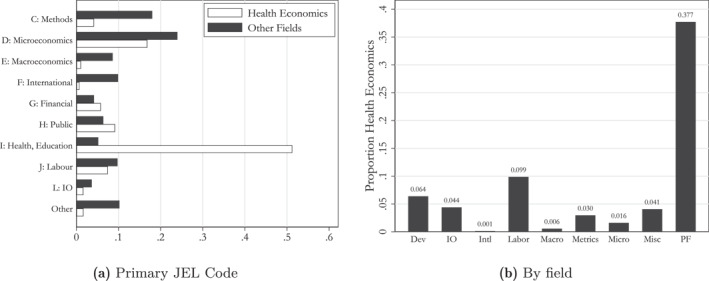
Comparison of classifications. The left chart shows the distribution of papers over different JEL codes depending on their classification as health economics papers. The right chart shows the distribution of papers over different fields (according to Angrist et al. 2020) depending on their classification as health economics papers.

We report the distribution of papers across different fields, conditional on our classification, in Figure 2. Figure 2a displays the distribution of health and non‐health papers over primary JEL codes. As expected, category I, “*Health, Education, and Welfare*,” is the most prominent group among health economics papers. Interestingly, the categories G “*Financial Economics*” and H “*Public Economics*” also show an over‐representation of health economics papers. The former is driven by category G22 “*Insurance, Insurance Companies, Actuarial Studies*” (92% of papers in the group), and the latter is driven by the categories H51 “*Government Expenditures and Health*” and H75 “*State and Local Government: Health, Education, Welfare, Public Pensions*”, which together account for 72% of papers in the group.

Figure 2b displays the proportion of health economics papers (according to our classification) within different fields of economics, as defined by Angrist et al. (2020). It is clear that the field “public finance” has by far the highest proportion; one‐third of papers classified as public finance are health economics papers according to our classifier. In all other fields, the proportion of health economics papers is below 10%, with particularly low proportions in Macroeconomics and International Economics.

In their bibliometric study of health economics, Wagstaff and Culyer (2012) identified health economics as having a health JEL code in any position—and they acknowledge that this is likely to generate false positives. We focus on the 300 papers over the 1969 to 2010 period that they identify as the most influential and study the classification of these papers in our data: the overlap is 73 papers. Among these 73 papers, the mean prediction is 0.87 for our author‐based classification and 0.89 for our journal‐based classification, suggesting that the vast majority would be classified as health economics by our classifier. Indeed, when we use our preferred combined classifier, all but 8 of the 73 papers are categorized as health economics. The exceptions are fairly revealing and include Acemoglu et al. (2001) on the colonial origins of development; Rodrik et al. (2004) on the role of institutions, geography, and trade in determining income levels; and Blau (1999) on the effect of income on child development.^1^

## 3 Novelty—Impact—Quality

### 3.1 Language Similarity Measures

In order to gain a deeper understanding of how innovative health economics papers have been, we adapt Kelly et al. (2021)'s methods for measuring technological innovations. The general idea of their approach is that high “novelty” of a patent is captured by low textual similarity to previous patents, while high “impact” is indicated by high textual similarity to future publications. “Quality” is defined as the difference or ratio of past and future similarity. Our analysis is based on the same principle but entails some adaptations to be suitable for research articles.

Kelly et al. (2021) rely on novel word combinations and their relative occurrence (tf‐idfs). We use sentence embeddings from the transformer‐based model, sentence‐t5‐xl (Ni et al. 2021). We compute vector representations for combined titles and abstracts of each paper, resulting in a 768‐dimensional vector with each dimension capturing a different aspect of the meaning of the text. We then calculate the cosine similarity (“sim”) between $v_{i}$, the vector representation of paper i, and ${\bar{u}}_{i}\left(a,b\right)$, an average vector representation of all papers published a to b years after paper i.

The backward similarity of a paper i is then given by the cosine similarity of the paper with the average vector representation of all papers published 5 to 1 year prior to the paper:

(1) $$BS_{i}=\text{sim}\left(v_{i},{\bar{u}}_{i}\left(-5,-1\right)\right).$$

The forward similarity compares the paper to all papers published 1–5 years after the paper.

(2) $$FS_{i}=\text{sim}\left(v_{i},{\bar{u}}_{i}\left(1,5\right)\right).$$

We further introduce the concept of “present similarity”, capturing the similarity to other papers published in the *same* year:

(3) $$PS_{i}=\text{sim}\left(v_{i},{\bar{u}}_{i}\left(0,0\right)\right).$$

Kelly et al. (2021) define “novelty” as the inverse of backward similarity. However, to avoid simply reflecting a paper's (dis‐)similarity to a generic economics paper, our “novelty” measure contrasts the backward similarity with the paper's similarity to contemporaneous papers. Thus, our indicator of *novelty* is defined as the difference between present and backward similarity:

(4) $$N_{i}=PS_{i}-BS_{i}.$$

Our *impact* measure is given by the difference between forward and present similarity:

(5) $$I_{i}=FS_{i}-PS_{i}.$$

Both measures range from −1 to 1. Using the transformation ${\overset{∼}{N}}_{i}=\left(N_{i}+1\right)/2$ and ${\overset{∼}{I}}_{i}=\left(I_{i}+1\right)/2$ yields a range from 0 to 1, where 1 represents maximum novelty. This allows us to define “quality” as the *product* of novelty and impact:

(6) $$Q_{i}={\overset{∼}{N}}_{i}\times {\overset{∼}{I}}_{i}.$$

This differs from Kelly et al. (2021), who define quality as the *ratio* between forward and backward similarity. We prefer our specification because ratio‐based measures tend to assign very high scores to observations with very low backward similarity, irrespective of their impact. Moreover, we argue that our measure is superior to one based on the *difference* between forward and backward similarity, as it ensures that a paper must score high on both novelty and impact to be considered of high quality.

A high‐novelty paper in this framework is one whose language differs substantially from earlier economics research but is still similar to that of contemporaneous work–possibly reflecting the introduction of a new topic, method, or policy concern. High impact, by contrast, reflects the degree to which the language used in a paper prefigures future discourse, indicating that it helped set an agenda. Papers scoring high on both dimensions–our definition of quality–are those that not only deviate from the past but also shape the future. Our method thus allows us to systematically identify paradigm‐shifting contributions without relying solely on retrospective citation‐based recognition.

Note that, due to how we calculate the measures based on language similarities, we have no comparison vectors for novelty in 1994 and for impact in 2023. Therefore, compared to our full sample starting in 1994 and ending in 2023, the time series for novelty starts in 1995 and ends in 2023, while the time series for impact starts in 1994 and ends in 2022. Overall, this means we cannot calculate novelty for 731 papers, impact for 2177 papers, and quality for a combined 2908 papers. This results in 35,208 papers for which we compute the quality measure.

### 3.2 Results

**FIGURE 3:**
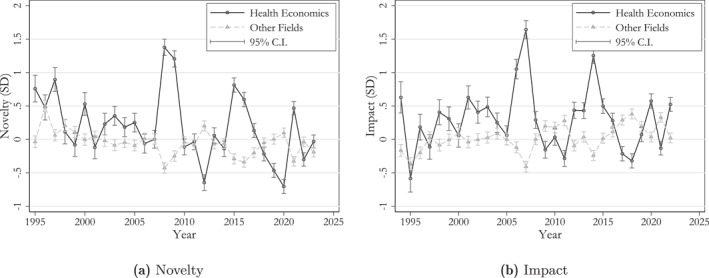
Novelty and impact ratings of papers in health economics and other fields. Estimates are based on the entire analysis sample with a computed quality measure (N=35,208) and include controls for journal fixed effects.

In Figure 3, we compare how the “novelty” and “impact” indicators evolve over time for health and non‐health publications. The figures suggest that health economics papers score higher on each indicator on average, but also that this advantage is concentrated in some peak years.

**FIGURE 4:**
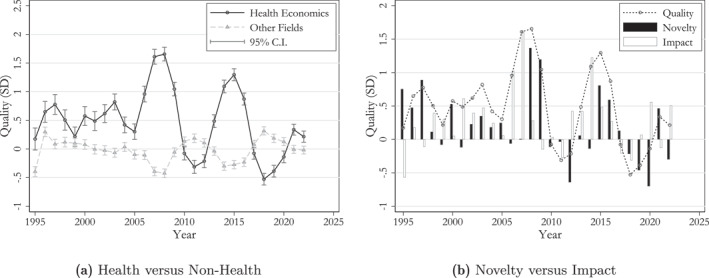
Quality ratings of papers in health economics and other fields. In Figure A4b, “novelty” and “impact” are measured in standard deviations of the “quality” score—in order to ensure that the annual average of the “quality” score is correctly decomposed. Estimates are based on the entire analysis sample with a computed quality measure (N=35,208) and include controls for journal fixed effects.

The corresponding graph for the combined “quality” measure is provided in Figure 4. Also, in this case, we observe a distinct advantage of health economics compared to other fields, particularly in certain years. To illustrate this, we provide the decomposition in Figure 4b. Interestingly, both major waves of increased “quality”—occurring in the 2006–2009 period and again in the 2014–16 period—are initially triggered by a surge in “impact”, followed by increased “novelty”.

Differentiating between the influence of “novelty” and “impact” reveals a relatively coherent pattern for each wave. Notably, the substantial uptick in “quality” during 2007–08, primarily attributed to heightened “impact,” appears largely driven by emerging literature emphasizing the significance of the early‐life period for adult outcomes. This is followed in 2008–09 by a surge in “novelty”, much of which arises from a cluster of studies examining the financial architecture of health systems. Finally, the 2014–16 wave is driven by high “impact” by research exploring the influence of financial incentives within health insurance schemes on the behaviors of both healthcare providers and patients, and by the application of new empirical methods to the field.

Starting with 2007–08 “impact” wave, papers like Bleakley (2007); Doyle Jr (2007); Ludwig and Miller (2007); Birchenall (2007); Black et al. (2007) and Chen and Zhou (2007) score high on impact, and all of them feature prominently in the review article on the “fetal origins hypothesis” published a few years later by Almond and Currie (2011). This strand of the literature seeks to understand how early‐life conditions—often beginning in utero or extending through early childhood—can have long‐lasting consequences for health, education, and labor market outcomes. A central insight, following from medical research such as that by David Barker, is that developmental processes in utero may be “programmed” by environmental factors including nutrition, disease exposure, and stress, with consequences that only manifest decades later. Economists have brought to this area a distinct emphasis on causal identification, exploiting quasi‐experimental variation to isolate the long‐term effects of early‐life interventions or shocks.

The studies cited above exemplify this agenda in a range of contexts. Bleakley (2007) evaluates the consequences of the early twentieth‐century campaign to eradicate hookworm in the American South, using regional variation in infection rates to show that treatment significantly improved educational attainment and long‐run earnings for children in highly infected areas. Similarly concerned with the consequences of health interventions in childhood, Ludwig and Miller (2007) investigate the *Head Start* program, exploiting a discontinuity in access driven by technical assistance offered to the poorest counties. Their findings suggest substantial reductions in child mortality and possible gains in educational attainment. In a related vein, Chen and Zhou (2007) examine the long‐run consequences of early‐life malnutrition, using a difference‐in‐differences design to compare birth cohorts exposed or unexposed to the Chinese famine of 1959–1961. They find lasting deficits in height and earnings among those exposed in infancy, highlighting the biological vulnerability of that developmental window.

Other papers focus on isolating the role of early adversity more broadly. Doyle Jr (2007) addresses the long‐term consequences of foster care placement, using the quasi‐random assignment of child protection investigators in Illinois as an instrument. He finds that children on the margin of placement often fare better when remaining with their families, especially among older children, suggesting that the foster care system may itself represent a developmental shock. Black et al. (2007) bring a unique twin design to the question of whether birth weight has causal effects on adult outcomes. Using Norwegian administrative data, they compare twins with different birth weights and find that even modest differences at birth predict disparities in adult height, IQ, education, and earnings—evidence that birth weight is more than just a proxy for unobserved background characteristics. Finally, Birchenall (2007) contributes to the theoretical foundations of the literature by modeling how health investments in early life can generate long‐term returns and shape the demographic transition, drawing on data spanning centuries of mortality change in England and Wales.

Together, these contributions reflect a broader reorientation of economic research on human capital formation—one that takes seriously the idea that the earliest stages of life, even those occurring before birth, can set trajectories that persist across the life course. By applying rigorous empirical methods to rich historical and administrative data, this literature has lent credence to the fetal origins hypothesis and expanded its implications far beyond health, into the domains of education, labor supply, and intergenerational mobility.

The continuation of the “quality” wave in 2008–09 is largely driven by a surge in “novelty,” much of which arises from a cluster of studies examining the financial architecture of health systems. Papers such as Flores et al. (2008); Grignon et al. (2008); Chi et al. (2008); Ariizumi (2008) and Wang et al. (2009) stand out within this surge, unified by a shared concern with how households interact with health care financing mechanisms—and how such interactions mediate access to care, exposure to risk, and ultimately health outcomes. These studies are notable not only for addressing policy environments outside the standard OECD focus but also for their conceptual and methodological ambition: despite working with constrained data or evolving institutional settings, they push the boundaries of how we measure the financial burden of illness, evaluate insurance design, and trace the behavioral consequences of different forms of public support. Whether analyzing coping strategies in India, the introduction of complementary insurance in France, out‐of‐pocket spending under universal coverage in Taiwan, or the comparative impacts of eligibility rules in long‐term care schemes, these contributions articulate a common agenda: to reframe financial protection as a dynamic, context‐sensitive process rather than a static insurance status. Taken together, they help to reorient the field toward the structural and distributional implications of health financing, laying conceptual groundwork that would shape later work on vulnerability, inequality, and the policy trade‐offs embedded in health system design.

Regarding the 2014–16 wave, the segment characterized by high “impact” is notably propelled by research exploring the influence of financial incentives within health insurance schemes on the behaviors of both healthcare providers and patients. Noteworthy examples include Clemens and Gottlieb (2014); Shigeoka (2014) and Chandra et al. (2014), which share a focus on causal identification and use natural experiments to explore how cost‐sharing and reimbursement rates influence both patient demand and provider supply. These studies reveal that both patients and providers are responsive to financial incentives, but in ways that vary across contexts, service types, and population groups. At the same time, they underscore the limits of price‐based mechanisms, as effects on health outcomes are often modest and responses among vulnerable populations may be constrained.

Conversely, the latter phase of this wave is driven by research high in “novelty”, with many contributions focusing on empirical methods (Jones et al. 2015; Kreif et al. 2015; McCarthy 2015; Armstrong 2015). These papers expand the analytical toolkit of health economics by addressing the limitations of standard models—whether in estimating full cost distributions, capturing dose–response relationships, aggregating multidimensional outcomes, or dealing with partial identification. Rather than incremental refinements, they offer conceptually distinct frameworks, often drawing on machine learning, simulation, or advances in econometric theory. Collectively, they reflect a broader ambition to close methodological gaps and enhance the robustness of empirical work in the field.

### 3.3 Relationship to Citations

A widely used measure of academic impact is the number of citations a paper generates. We assess the performance of our “novelty”, “impact”, and “quality” measures by examining how well they predict citations. First, we consider the general correlation patterns between these indicators and citations. Results from simple OLS regressions, where citations normalized by publication year are used as the dependent variable, are presented in Table 3. In the left panel, we estimate the raw correlations between our three indicators and citations. A one‐standard‐deviation increase in “novelty” is associated with a 3.8% increase in citations, while a one‐standard‐deviation increase in “impact” is associated with a 4.3% increase in citations. The difference between the coefficients is not statistically significant.

**TABLE 3:** Prediction of citations.

	Unconditional				Conditional on journal			
	(1)	(2)	(3)	(4)	(5)	(6)	(7)	(8)
Novelty	0.0384***		0.0777***		0.0749***		0.1386***	
	(0.012)		(0.013)		(0.011)		(0.013)	
Impact		0.0431***	0.0809***			0.0531***	0.1239***	
		(0.012)	(0.013)			(0.011)	(0.013)	
Quality				0.0803***				0.1333***
				(0.012)				(0.012)
Journal F.E.					✓	✓	✓	✓
Year F.E.					✓	✓	✓	✓
*N*	35,208	35,208	35,208	35,208	35,208	35,208	35,208	35,208

*Note:* OLS regressions of year‐normalized citation counts on the main analysis sample. Independent variables are measured in standard deviations. Standard errors in parentheses. In Figures A2 and A3 in Appendix C, we report binned scatter plots for the univariate regressions in this Table.

*p<0.05.

**p<0.01.

^***^ p<0.001.

However, “novelty” and “impact” are negatively correlated, with a correlation coefficient of −0.49, which biases OLS estimates. Consequently, when we include both indicators in a single regression specification, the estimated coefficients almost double. In the fourth column, we estimate the coefficient of the combined “quality” indicator, which suggests that a one‐standard‐deviation increase in “quality” is associated with 8% more citations.

In columns (5) to (8), we estimate the correlation between our indicators and citations, conditional on the journal and the year in which the papers were published. This changes the interpretation of the estimates, as the three indicators may affect the likelihood of being published in a particular journal. Indeed, we find that including journal and year fixed effects substantially magnifies the estimated coefficients, with factors ranging from 1.3 to 1.9.

While the language similarity measures align positively with citations, they capture dimensions of scientific quality that are conceptually distinct from traditional bibliometrics. Citations also reflect recognition and are, for example, influenced by author and journal prominence. Instead, the language similarity measures rely on textual embeddings of the content of the abstracts. In particular, the novelty measure reflects how far a paper departs from prior work, regardless of whether that work is cited. In contrast, impact captures the paper's semantic similarity to future research, regardless of whether citations are formally made. Additionally, citations may accumulate slowly and are truncated for more recent articles in our sample. We try to correct for this circumstance by normalizing within years, but the language similarity measures may allow us to better identify influential work, particularly before citations have fully materialized.

### 3.4 Health Economics Versus Non‐Health Papers Performance

We next turn to the question of whether health economics papers perform differently compared to non‐health papers. This is of interest since there is a common conception that fields close to life sciences (such as health economics) tend to generate more citations than other fields. If this is the case, it would, for example, rationalize the trend we observe in Figure 1 to the extent that journal editors put increasing weight on citations.

Results are presented in Table 4, which has the same structure as Table 3 but includes interactions with our paper classifier. We can clearly refute the notion that health economics is treated more favorably than other fields. The intercept for health economics papers is lower than for other papers; however, this difference diminishes when we condition on the outlet. Additionally, we find that health economics papers are systematically less rewarded for “novelty”, “impact”, and “quality”. Specifically, papers classified as health economics are associated with significantly lower coefficients for all three indicators. This finding remains remarkably robust even when journal fixed effects are included.

**TABLE 4:** Prediction of citations: Health versus non‐health.

	Unconditional				Conditional on journal			
	(1)	(2)	(3)	(4)	(5)	(6)	(7)	(8)
Health	−0.4393***	−0.4368***	−0.4762***	−0.4770***	−0.0524	−0.0474	−0.0707	−0.0710
	(0.028)	(0.028)	(0.030)	(0.030)	(0.047)	(0.047)	(0.048)	(0.048)
Novelty	0.0585***		0.1428***		0.0797***		0.1627***	
	(0.013)		(0.015)		(0.013)		(0.015)	
Health × novelty	0.0028		−0.0596*		−0.0217		−0.0860***	
	(0.029)		(0.032)		(0.030)		(0.033)	
Impact		0.0770***	0.1542***			0.0583***	0.1480***	
		(0.013)	(0.015)			(0.013)	(0.015)	
Health × impact		−0.0452	−0.0914***			−0.0244	−0.0872***	
		(0.030)	(0.033)			(0.030)	(0.034)	
Quality				0.1508***				0.1579***
				(0.014)				(0.014)
Health × quality				−0.0763***				−0.0881***
				(0.027)				(0.028)
Journal F.E.					✓	✓	✓	✓
Year F.E.					✓	✓	✓	✓
*N*	35,208	35,208	35,208	35,208	35,208	35,208	35,208	35,208

*Note:* OLS regressions of year‐normalized citation counts on the main analysis sample. Independent variables are measured in standard deviations. Standard errors in parentheses.

*p<0.05.

**p<0.01.

^***^ p<0.001.

One potential concern is that changes over time in how authors frame their work could influence this classification. However, since our health economics identifier is based on machine learning applied to abstracts and metadata—not on self‐labeling—we believe it is relatively robust to such strategic shifts.^2^

### 3.5 COVID‐19 and the Evolving Position of Health Economics

The COVID‐19 pandemic represents a profound and immediate shock to public health systems and societies at large, and it likely affected the field of health economics more directly than most other areas of economics. In response, a large number of economics papers focused specifically on the pandemic were published in a very short period of time. This rapid proliferation of COVID‐related research prompts the question of whether the recent increase in the prominence of health economics, documented in earlier sections, is primarily driven by this literature—or whether it reflects a broader and more sustained trend.

To investigate this, we identify subfields within health economics using unsupervised topic modeling, as described in Appendix D. One resulting cluster, centered on pandemics and public health interventions, spans both COVID‐19 and pre‐COVID literature. Importantly, this subfield stands out in the data: as shown in Appendix Table A2, papers within this category consistently outperform others on all our performance measures—citations, novelty, impact, and the combined quality index. This motivates a closer examination of whether the COVID‐19 papers alone are responsible for these results, or whether the broader pandemic literature has long played a distinctive role within health economics.

**FIGURE 5:**
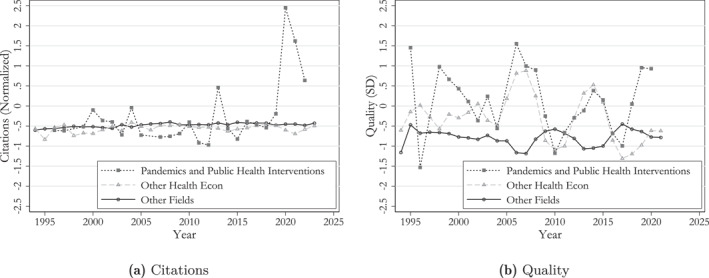
Quality ratings of papers in health economics and other fields. In Figure A4b, “novelty” and “impact” are measured in standard deviations of the “quality” score—in order to ensure that the annual average of the “quality” score is correctly decomposed. Estimates are based on the entire analysis sample with a computed quality measure (N=35,208) and include controls for journal fixed effects.

To address this, we classify all papers into three categories: (1) COVID, pandemics, and vaccines; (2) other health economics; and (3) all other economics. In Figure 5, we compare citation counts and quality scores across these categories.

**FIGURE A4:**
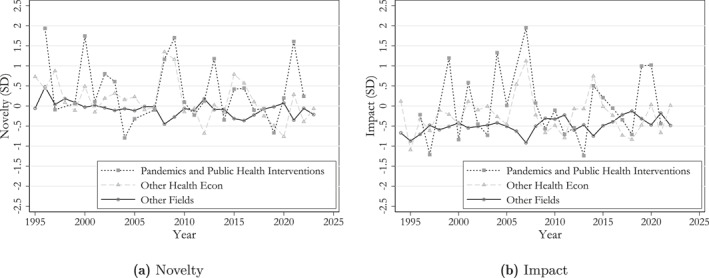
Novelty and impact ratings of papers in health economics and other fields. Estimates are based on the entire analysis sample with a computed quality measure (N=35,208) and include controls for journal fixed effects.

Clearly, papers published during the pandemic have disproportionately contributed to the good performance in citations. Apart from a spike in citations in 2013, driven by two highly cited papers (Fenichel 2013; Rassy and Smith 2013), the years of the COVID‐19 pandemic dominate this metric. However, when it comes to the “quality” indicator, the pandemic period does not drive the results as strongly: notable peaks also occur around 1999 and 2007. These peaks can be attributed to contributions such as Mullahy (1999) studying flu shots and Montalvo and Reynal‐Querol (2007) investigating the impact of war refugees on the spread of malaria. In Appendix Figure A4 we present a breakdown of “quality” by the “novelty” and “impact” components.

The spike in citations of the health economics subfield studying “Pandemics and Public Health Interventions” highlights how the salience of a topic can influence short‐term citation patterns. In contrast, our language similarity measures show only a modest rise during the pandemic period, similar to earlier waves. This suggests that the COVID‐19 papers were not systematically more novel or impactful in shaping research than previous work within the subfield, at least in terms of textual content. Additionally, this provides supportive evidence that the pandemic did not drive the overall increase in “health economics” relative importance within the economics literature we documented in the previous sections.

## 4 Conclusion

The evolving role of health economics within general economic research and publishing over the past 30 years marks a significant transformation from a niche field to a mainstream area of interest. Historical contributions from seminal scholars like Arrow (1963), Newhouse (1970), and Grossman (1972) laid foundational stones, yet health economics rarely featured in prestigious general‐interest economics journals. However, recent decades have witnessed a dramatic shift, with health economists increasingly contributing to and gaining recognition in broader economic discourse. This trend is vividly illustrated by the rising proportion of health economics papers in top‐tier and general‐interest journals, and the substantial growth in health economics research in related fields such as labor, development, and public economics.

Our analysis identifies several key factors behind the rising prominence of health economics in academic publishing. We utilize advanced bibliometric methods, leveraging natural language processing (NLP) techniques with RoBERTa to accurately classify health economics papers. Our findings suggest that the integration of health economics into mainstream economics is driven primarily by innovative, high‐quality research rather than mere conformity to existing norms. By adapting Kelly et al. (2021)'s method to evaluate the novelty, impact, and quality of academic papers, we demonstrate that health economics papers consistently exhibit higher quality, indicating a substantial contribution to the field's evolution. This shift not only underscores the field's growing importance but also highlights its potential to influence and enrich general economic research.

Our analysis identifies two distinct waves in the “quality” ratings of health economics research, each driven by different factors: the 2007–09 wave was initially largely influenced by “impact”, highlighting studies on early‐life health influences; later, it was characterized by “novelty,” focusing on the financial architecture of health systems. The 2014–16 wave was driven by high “impact” research on behavioral responses to financial incentives in health insurance schemes and by the application of new empirical methods to the field. We further find a significant positive correlation between citations and our quality ratings. Our analysis also shows that health economics papers are systematically less rewarded by citations for “novelty”, “impact”, and “quality” compared to papers from other fields of economics. Finally, our study reveals that much of the recent citation boost is attributable to pandemic‐related research, particularly during 2020 and 2021. While our “novelty” and “impact” measures also increase during this period, the rise is less pronounced and not exceptional relative to earlier waves of research activity, suggesting that the surge in citations likely reflects topical salience more than a dramatic shift in research quality.

Classifying a large corpus of publications spanning several decades presents inherent challenges. A key concern in our case is that we rely on the *published* versions of papers, which may reflect strategic framing choices by authors or editorial influences arising from the review process. As the status of health economics has evolved over time—a development we document in this paper—such shifts may affect how papers are framed, introducing potential confounding in the classification. This is an important caveat to bear in mind when interpreting our results.

Nonetheless, we contend that our approach is likely more robust to such confounding than existing alternatives. First, we apply the same algorithm uniformly across all time periods, making our classification less sensitive to shifting perceptions of what constitutes the field of health economics. Second, the author‐based component of our classification helps mitigate the risk of strategic framing: papers authored by individuals classified as health economists will still be labeled as health economics, even if intentionally framed to appear otherwise (and vice versa). Third, reframing an abstract to alter the perception of a paper requires more effort than optimizing JEL codes or keywords, making it less prone to manipulation. Taken together, these features suggest that while limitations remain, our method offers a comparatively robust strategy for field classification in bibliometric research.

## Conflicts of Interest

The authors declare no conflicts of interest.

## Figures and Tables

**FIGURE A1:**
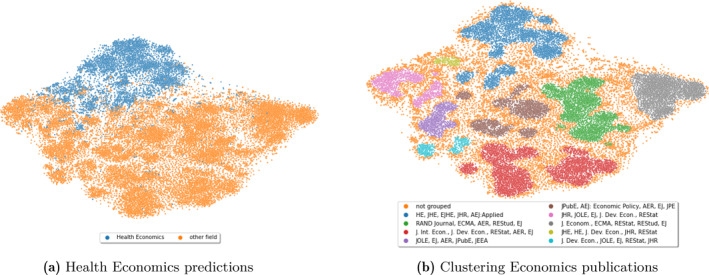
Locating health economics within economics. This Figure plots the dimensionality‐reduced sentence‐t5‐xl text embeddings for all 38,116 papers in our sample. To reduce the dimensions of the 768‐dimensional sentence‐t5‐xl vectors, we employ a combination of PCA (reducing 768 dimensions to 50) and t‐SNE (reducing 50 dimensions to 2). The nine distinct clusters in Panel (b) result from first clustering the two‐dimensional text representations into 24 distinct clusters, which were then combined if they shared the most common journal.

**FIGURE A2:**
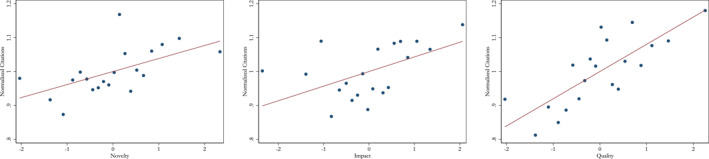
Binned scatter plots of text‐based measures on citations. This Figure shows binned scatter plots with 20 bins of our text‐based measures and normalized citations for each health economics paper in our sample. The regression fit in the plots represents the regressions reported in 3 Columns (1), (2), and (4), without journal fixed effects.

**FIGURE A3:**
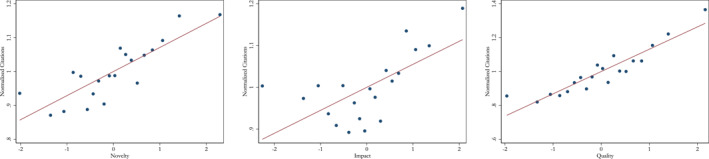
Binned scatter plots of text‐based measures on citations with journal fixed effects. This Figure shows binned scatter plots with 20 bins of our text‐based measures and normalized citations for each health economics paper in our sample. The regression fit in the plots represents the regressions reported in 3 Columns (5), (6), and (8), including journal fixed effects.

## Data Availability

The data that support the findings of this study are available from the corresponding author upon reasonable request.

## Endnotes

^1^
In Appendix B, we provide a further type of validation, showing visualizations of our paper classification.

^2^
For instance, if authors in earlier periods had incentives to avoid labeling their papers as health economics (e.g., due to perceived biases in general‐interest journals), this would affect our classification only to the extent that it altered the textual and contextual features of their papers. Even then, our author‐based classification provides an additional safeguard against such strategic framing.

## Appendix A Classifier Training

We trained a classifier based on RoBERTa‐large using both the author‐ and the journal‐based classification. The inputs for the classifiers are the concatenated titles and abstracts of the publications in our training samples. The first step of the transformer architecture consists of turning the raw text into a numerical representation, so‐called text embeddings, via an encoder. These text embeddings can then be fed into the neural network structure of RoBERTa. On top of RoBERTa, we add a linear layer that reduces the dimensionality of the output from 1024 to 2. This allows us to interpret the raw output as relative weights, whether a paper is a health economics publication or belongs to a different field. Finally, we add a sigmoid layer, which applies a logistic function to the raw outputs, normalizing them between 0 and 1, thereby allowing us to interpret the final output of the classifier as probabilities. For training, we split our data into subsets with proportions of 0.7, 0.2, 0.1 for the training, validation, and testing data, respectively. We combined the two classification methods via ROC curves to enhance robustness and performance. The combined classifier was then used to predict health economics papers in a total sample of 38,116 papers. This total includes the two training datasets and papers within category (2) that could not be labeled for training due to having either unlabeled authors or combinations of health and non‐health authors.

## Appendix B Visualization Via Dimensionality Reduction

Figure A1 provides an alternative type of validation, showing visualizations of our paper classification. We use a combined procedure of Principal Component Analysis (PCA) and t‐distributed Stochastic Neighbor Embedding (t‐SNE) to reduce the dimensionality of the sentence‐t5‐xl text embeddings. In Figure A1a, our predictions of Health Economics papers form a relatively homogeneous group within this mapping of economics papers. To provide some additional context, we report the result of hierarchical clustering (cf. Campello et al. 2013) of the dimensionality‐reduced text embeddings. Furthermore, we highlight the 5 most common journals in each of these clusters. This further emphasizes the relationship between health economics and labor, development, and public economics.

## Appendix C Additional Tables and Figures

In Figures A2 and A3, we provide additional visualizations of the regressions shown in Table 3.

## Appendix D Subfield Analysis

In addition to the supervised field classification described in Section 2.2, we use unsupervised learning in the form of topic modeling to identify subfields within health economics. Specifically, we employ BERTopic, a method proposed by Grootendorst (2022). BERTopic uses transformer‐based sentence embedding models and non‐linear dimensionality reduction methods^3^ to produce dense topical clusters of text. The topic representations, that is the “names” of the topics, are found as the most important words for each cluster via term frequency‐inverse document frequency (tf‐idf). In our implementation of BERTopic, we use sentence‐t5‐xl (Ni et al. 2021) as the sentence‐embedding model and require every topic/health economics subfield of at least 100 publications. In Table A1 we present the resulting subfields of health economics. Note that subfield m: health insurance and long‐term care benefits combines all papers into a single subfield that were not grouped into any other subfield.

**TABLE A1:** Subfield overview.

Subfield	Keywords	No.	Percent
A: Healthcare spending and economic burden	Care costs, healthcare costs, cost effectiveness, costs, cost, treatment, economic burden, hrqol, health care, healthcare	273	3.1%
B: Early‐life health and birth outcomes	Birth outcomes, child health, birth weight, health outcomes, children born, early life, infant health, impacts, outcomes, infant mortality	460	5.3%
C: Substance use, addiction, and tobacco control	Tobacco control, alcohol use, cigarettes, addiction, smokers, smoking, effects, tobacco, alcohol consumption, cigarette	655	7.5%
D: Cost‐effectiveness and health intervention evaluation	Cost effectiveness, effectiveness analyses, effectiveness analysis, methods, effectiveness, clinical trial, clinical trials, economic evaluation, economic evaluations, cost data	253	2.9%
E: Pandemics and public health interventions	19 pandemic, pandemic, epidemics, epidemic, outbreak, covid 19, outbreaks, interventions, vaccination rates, public health	289	3.3%
F: Health utility measurement and EQ‐5D valuations	eq 5d, quality life, health states, health state, values, adjusted life, 5d 5l, valuations, health related, results	693	8.0%
G: Health expenditures and service utilization	Health outcomes, health insurance, health expenditure, health expenditures, health care, health spending, national health, health services, catastrophic health, healthcare	184	2.1%
H: Health insurance markets and policy reform	Health insurance, insurance coverage, insurance markets, insurance market, health plan, private health, health care, insurance, affordable care, medicare	588	6.8%
I: Health shocks, retirement, and aging	Health retirement, health status, health shocks, mental health, retirement age, poor health, health, retirement, early retirement, pension	253	2.9%
J: Socioeconomic inequalities in health	Inequalities health, health inequalities, inequality health, income health, health inequality, health income, income inequality, health status, income related, socioeconomic status	256	2.9%
K: Pharmaceutical pricing and market regulation	Price regulation, reference pricing, pricing, pharmaceutical, prices, prescription drugs, pharmaceutical firms, pharmaceutical industry, pharmaceuticals, price	277	3.2%
L: Hospital quality and medicare outcomes	Quality care, hospitals, health care, medicare, healthcare, outcomes, hospital, medical, health, rates	1061	12.2%
M: Health insurance and long‐term care benefits	Health care, healthcare, health insurance, health, medical, outcomes, insurance, benefits, long term, age	3444	39.6%

*Note:* The table presents subfields of health economics based on topic models. The subfield names were suggested by GPT‐4‐turbo based on the keywords of each topic. The keywords of each topic represent the most descriptive words for a topic compared to all other topics based on TF‐IDFs and are computed by Grootendorst (2022). We also report the number of papers and their percentage shares among all 8686 health economics papers.

In Table A2 we compare the performance of the different subfields within health economics, regressing the indicators on subfield categories and journal and year fixed effects. We use the largest subfield—l: hospital quality and medicare outcomes—as the reference category. Notably, the estimates for the four indicators are in great agreement for categories A, B, E, F, G, H, I, and M. Categories d: cost‐effectiveness and health intervention evaluation and k: pharmaceutical pricing and market regulation on the other hand are noted for scoring high on citations, but low on the other indicators. This possibly reflects the fact that these two subfields are the most applied ones, often targeting an audience of practitioners rather than the academic community. In Table A3 we present the corresponding results for a specification without journal and year fixed effects, and the results are qualitatively very similar.

**TABLE A2:** Subfield‐specific results.

	Citations	Novelty	Impact	Quality
	(1)	(2)	(3)	(4)
A: Healthcare spending and economic burden	0.1650**	0.0279	0.1137**	0.1383**
	(0.083)	(0.056)	(0.057)	(0.059)
B: Early life health and birth outcomes	0.4011***	0.0985**	0.0769*	0.1717***
	(0.066)	(0.045)	(0.045)	(0.047)
C: Substance use, addiction, and tobacco control	0.1211**	−0.0134	0.0225	0.0074
	(0.057)	(0.039)	(0.039)	(0.041)
D: Cost‐effectiveness and health intervention evaluation	0.2913***	−0.1417***	−0.0270	−0.1698***
	(0.080)	(0.054)	(0.055)	(0.057)
E: Pandemics and public health interventions	1.2539***	0.6358***	0.2712***	0.8976***
	(0.084)	(0.057)	(0.058)	(0.060)
F: Health utility measurement and EQ‐5D valuations	0.3297***	0.0040	0.0717*	0.0732*
	(0.057)	(0.039)	(0.039)	(0.041)
G: Health expenditures and service utilization	0.3715***	0.1031*	0.0963	0.1946***
	(0.092)	(0.063)	(0.064)	(0.066)
H: Health insurance markets and policy reform	−0.0288	0.0541	−0.0501	0.0047
	(0.059)	(0.040)	(0.041)	(0.042)
I: Health shocks, retirement, and aging	0.4882***	0.0176	0.1215**	0.1349**
	(0.081)	(0.055)	(0.056)	(0.058)
J: Socioeconomic inequalities in health	0.3660***	0.0111	0.0384	0.0477
	(0.079)	(0.054)	(0.055)	(0.056)
K: Pharmaceutical pricing and market regulation	0.2284***	−0.1086**	−0.1809***	−0.2860***
	(0.078)	(0.053)	(0.053)	(0.055)
M: Health insurance and long‐term care benefits	0.1679***	0.0759***	0.0275	0.1012***
	(0.041)	(0.028)	(0.028)	(0.029)
Journal F.E.	✓	✓	✓	✓
Year F.E.	✓	✓	✓	✓
*N*	8031	8031	8031	8031

*Note:* OLS regressions of year‐normalized citation counts and quality indicators on the main analysis sample of health economics publications. The reference category is l: hospital quality and medicare outcomes. Standard errors in parentheses.

^*^ p<0.05.

^**^ p<0.01.

^***^ p<0.001.

**TABLE A3:** Subfield‐specific results.

	Citations	Novelty	Impact	Quality
	(1)	(2)	(3)	(4)
A: Healthcare spending and economic burden	−0.0714	−0.0948	0.1316**	0.0334
	(0.082)	(0.065)	(0.063)	(0.071)
B: Early life health and birth outcomes	0.5375***	0.0312	0.0890*	0.1164**
	(0.067)	(0.053)	(0.051)	(0.058)
C: Substance use, addiction, and tobacco control	0.1445**	0.0383	0.0479	0.0836
	(0.060)	(0.047)	(0.045)	(0.051)
D: Cost‐effectiveness and health intervention evaluation	0.1199	−0.0595	0.0082	−0.0540
	(0.083)	(0.065)	(0.063)	(0.071)
E: Pandemics and public health interventions	1.2335***	0.5901***	0.1811***	0.7642***
	(0.087)	(0.068)	(0.066)	(0.075)
F: Health utility measurement and EQ‐5D valuations	0.1749***	0.0496	0.0958**	0.1419***
	(0.059)	(0.046)	(0.045)	(0.051)
G: Health expenditures and service utilization	0.2513***	0.1976***	0.1349*	0.3262***
	(0.097)	(0.076)	(0.074)	(0.083)
H: Health insurance markets and policy reform	0.1035*	0.0622	−0.1061**	−0.0419
	(0.062)	(0.049)	(0.047)	(0.053)
I: Health shocks, retirement, and aging	0.4502***	−0.0203	0.1073*	0.0834
	(0.085)	(0.067)	(0.065)	(0.073)
J: Socioeconomic inequalities in health	0.3147***	0.1415**	0.0386	0.1773**
	(0.083)	(0.065)	(0.063)	(0.071)
K: Pharmaceutical pricing and market regulation	0.3081***	−0.1529**	−0.1488**	−0.2989***
	(0.081)	(0.064)	(0.062)	(0.070)
M: Health insurance and long‐term care benefits	0.2038***	0.0509	0.0447	0.0931**
	(0.042)	(0.033)	(0.032)	(0.037)
*N*	8031	8031	8031	8031

*Note:* OLS regressions of year‐normalized citation counts and quality indicators on the main analysis sample of health economics publications. The reference category is l: hospital quality and medicare outcomes. Standard errors in parentheses.

^*^ p<0.05.

^**^ p<0.01.

^***^ p<0.001.
